# Students’ Perceptions of Educational Climate in a Spanish School of Dentistry Using the Dundee Ready Education Environment Measure: A Longitudinal Study

**DOI:** 10.3390/dj8040133

**Published:** 2020-12-07

**Authors:** Alba María Hernández-Crespo, Paula Fernández-Riveiro, Óscar Rapado-González, Ángela Aneiros, Inmaculada Tomás, María Mercedes Suárez-Cunqueiro

**Affiliations:** 1 Department of Surgery and Medical-Surgical Specialties, Medicine and Dentistry School, Universidade de Santiago de Compostela (USC), 15782 Santiago de Compostela, Spain; albahzcrespo@gmail.com (A.M.H.-C.); oscar.rapado@rai.usc.es (Ó.R.-G.); angela_aneiros@hotmail.com (Á.A.); 2 Department of Psychiatry, Radiology Public Health, Nursing and Medicine, Universidade de Santiago de Compostela (USC), 15782 Santiago de Compostela, Spain; 3 Health Research Institute of Santiago (IDIS), 15782 Santiago de Compostela, Spain; 4 Oral Sciences Research Group, Health Research Institute of Santiago (IDIS), 15782 Santiago de Compostela, Spain

**Keywords:** educational climate, dental students, DREEM scale, dental education, dentistry

## Abstract

Background: Educational Climate (EC) may determine teacher and student behaviour. Our aim was to evaluate EC longitudinally in a period of ‘curricular transition’ from traditional (teacher-centred learning) to Bologna curricula (interactive student-centred learning). Methods: The ‘Dundee Ready Education Environment Measure’ (DREEM) questionnaire was completed by 397 students from a Spanish School of Dentistry. Students’ perception was assessed in different courses and academic years. Results: The overall EC scale average was 115.70 ± 20.20 (57.85%) and all domain values showed a percentage > 52%, which were interpreted as ‘positive and acceptable’. The EC mean was: 118.02 ± 17.37 (59.01%) for 2010–2011; 116.46 ± 19.79 (58.23%) for 2013–2014; 115.60 ± 21.93 (57.80%) for 2014–2015; 112.02 ± 22.28 (56.01%) for 2015–2016, interpreted as ‘more positive than negative EC’. The worst Learning domain scores corresponded to later academic years and may reflect the Bologna curriculum’s more intensive clinical training involving greater responsibility and self-learning. Conclusions: EC and its domains were perceived more positively than negatively. The Social domain was the most positively evaluated, while the Learning domain was the worst.

## 1. Introduction

The educational environment is defined as students’ perceptions of their influences and pressures, and how this perception is aligned with the curriculum’s educational aims [1]. The perception of the educational environment is considered as the ‘Educational Climate’ (EC). The term EC has been described by Genn et al. [2] as “the expression of the educational environment and the academic curriculum”. Thus, EC is considered “everything that is happening in the classroom, in a department, in the faculty or the university” [2]. Students’ perceptions regarding EC are influenced by aspects such as learning outcomes, teacher skills, learning resources, learning and teaching approaches, assessment procedures, timetabling, student support, facilities, classrooms, group size, and atmosphere [3]. In addition, EC encourages teacher attitudes, and student achievements. Thus, EC has a significant impact on academic success, and on professional development [2,3,4,5] as well as being critical to students’ personal and social well-being. Moreover, it is established the EC affects not only students, but also school staff, and even curriculum designers and administrative personnel [5]. The EC profile represents an opportunity to ascertain the strengths and weaknesses of an institution, thus enabling comparative analysis within or between institutions in order to foster change and improvement in the educational process. Universities should continuously evaluate the EC of their classes, departments, and schools to detect problems and implement corrective measures.

Several qualitative and quantitative methodologies have been used to evaluate students’ perception of EC [6,7,8,9]. In academic healthcare institutions, one of the most widely used and reliable instruments is the ‘Dundee Ready Educational Environment Measure’ (DREEM) [10,11]. The DREEM was developed by a Delphi panel including 100 educators specializing in healthcare disciplines from 20 countries, and 1000 students. Currently, the DREEM questionnaire exists in different languages and has been widely used to assess EC in Dentistry Schools worldwide [9,12,13,14,15,16,17]. The DREEM scale is a universal tool, applicable regardless of national development level. 

As a result of the Bologna reform process in the European Union [18], it was of great importance to determine student perception of EC during the ‘curricular transition’ period. The education reform derived from the European convergence process entailed an educational philosophy that focused more on the student being the learner, than the teacher being a person who teaches [19,20,21]. In other words, this educational reform involved a transition from traditional teacher-centred learning to interactive student-centred learning. Seeing as no scientific literature was available regarding this curricular transition in Dentistry Schools, we decided to measure EC using the DREEM to determine how students perceived this change and to detect problem areas. Thus, the purpose of this study was to longitudinally evaluate EC for undergraduate dental students in a period of ‘curricular transition’ brought on by the Bologna reform process.

## 2. Materials and Methods 

### 2.1. Study Group

**Figure 1:**
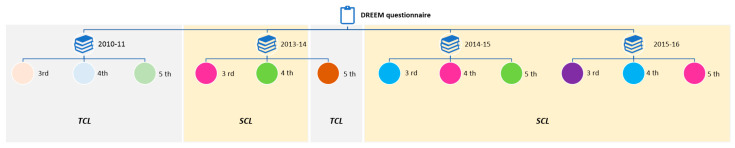
Workflow of study design showing the application of DREEM questionnaire on the different academic years and courses during ‘curricular transition’. Each colour represents a different student’s dental group indicating the same colour the study follow-up. Abbreviations: TCL, teacher-centred learning; SCL, student-centred learning.

We carried out a prospective longitudinal study applying the DREEM questionnaire to undergraduate dental students from the Medicine and Dentistry School, Universidade de Santiago de Compostela (USC), Spain. Ethical approval for the study was obtained from the Universidade de Santiago de Compostela. The students in the sample were from 3rd, 4th, and 5th year courses. Before participating in the survey, students were informed regarding the data processing characteristics, the importance of voluntary participation, and the anonymity of the process. The average questionnaire completion time was approximately 7 min. Questionnaires were distributed to students at the end of several academic years (2010–2011, 2013–2014, 2014–2015, and 2015–2016), which had different curricular configurations: in the 2010–2011 academic year, all courses were taught using a teacher-centred approach, in the 2013–2014 academic year, the 3rd and 4th year courses used a student-centred approach, while 5th year courses used a teacher-centred approach. Finally, in the last two academic years (2014–2015, 2015–2016), all courses used a student-centred approach (Figure 1). 

### 2.2. Data Collection

The instrument used in this study was the DREEM questionnaire (Supplementary Table S1) which consists of 50 items, grouped into five domains: D1. Students’ perception of learning (Learning), D2. Students’ perception of teachers (Teachers), D3. Students’ academic self-perception (Academic), D4. Students’ perception of the atmosphere at the centre (Atmosphere) and D5. Students’ social self-perception (Social). Each of these items was given a score based on a Likert scale with 5 options ranging from 4 to 0 (4 = strongly agree, 3 = agree, 2 = uncertain, 1 = disagree, 0 = strongly disagree). Nearly all DREEM items include positive statements, except items 4, 8, 9, 17, 25, 35, 39, 48, and 50, which are negative, thus their scores are reversed [10,17]. The mean scores for the different items, domains, and EC were interpreted according to the criteria established by McAleer and Roff et al. [10,22]. Therefore, the items with an average value of ≥3.50 were considered to be “educational aspects of excellence”; those between 3.01 and 3.49 were considered to be “positive educational aspects”; those with average values between 2.01 and 3.00 were considered to be “educational aspects that could be improved”; those ≤2.00 were defined as “problematic educational areas, which should be examined more exhaustively later”. The DREEM questionnaire was validated for the Spanish language in 2014 by Tomas et al. [23].

The DREEM scale provides results for each item, for each domain (the sum of the scores of the corresponding items) and total EC score (the sum of the scores of each domain). The maximum possible scores for the different domains are: Learning Perception: 48; Teacher Perception: 44; Academic Perception: 32; Atmosphere Perception: 48 and Social: 28. The maximum score for EC is 200. The data can be expressed as percentages of maximum scores in the respective subscale or the overall scale. Therefore, in relation to the general interpretation of the scale, the higher the score (or percentage) obtained in the different parameters, the more positive perception about the aspect evaluated. 

### 2.3. Statistical Analysis

The data obtained were processed with the PASW Statistics program (SPSS version 2.1) for Windows. The data in the overall assessment of EC, for each domain, and each questionnaire item were expressed as averages. The data in the overall assessment of the EC and for each domain were also expressed as percentages in relation to the maximum score [10]. Non-parametric tests, such as the Kruskal–Wallis test and the Mann–Whitney U test, were used for comparing ordinal EC variables, domains, and items between courses and academic years. Significance level was considered as *p* ≤ 0.05. In the case of multiple comparisons between academic years, the Bonferroni correction was applied, establishing a value of *p* < 0.008 as significant. In the case of multiple comparisons between the teaching courses in different academic years, the Bonferroni correction was applied, establishing a *p*-value < 0.016 as significant.

## 3. Results

### 3.1. Description of the Study Group

A total of 397 (70%) dentistry students completed the DREEM questionnaire. There were one hundred and eighteen (29.7%) males and two hundred and seventy-five (69.3%) females (gender data were unavailable for four subjects). The average age was 23.19 ± 4.62 years. Regarding the different courses, there were 117 students in the 3rd course, 119 in the 4th course and 161 in the 5th course.

### 3.2. Global Analysis of ‘Educational Climate’

**Figure 2:**
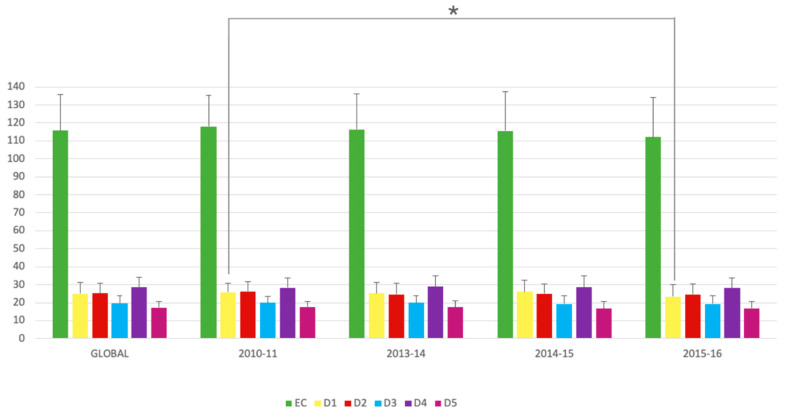
Means of scores and standard deviations for ‘Educational Climate’ and domains in the Dundee Ready Education Environment Measure (DREEM) questionnaire by academic years. * *p*-Value < 0.05.

The overall EC mean was 115.70 ± 20.20 (57.85%), which was interpreted as “more positive than negative EC”. According to domain values, all were interpreted as “positive and acceptable”. The mean obtained for Learning domain was 25.12 ± 6.04 (52.12%), for Teacher domain was 25.15 ± 5.79 (57.15%), for Academic domain was 19.65 ± 4.11 (61.40%), for Atmosphere domain was 28.44 ± 5.87 (59.25%) and for Social domain was 17.21 ± 3.59 (61.49%) (Table 1 and Figure 2). Social domain was the best-evaluated domain, within the range of “positive and acceptable”. Learning domain was the worst-evaluated domain, within the range of “problematic educational aspect”. Regarding the total of items derived from the four surveys of each academic year, with 50 items each (total of 200 items), 23.5% were within the range of “problematic educational aspects” (47 items), 68.5% in the range of “educational aspects that could be improved” (137 items) and 8% were “positive educational aspects” (16 items). None was found in the range of “educational aspects of excellence”.

### 3.3. Analysis of ‘Educational Climate’ by Academic Year

The mean EC by academic year were as follows: 118.02 ± 17.37 (59.01%) for 2010–2011; 116.46 ± 19.79 (58.23%) for 2013–2014; 115.60 ± 21.93 (57.8%) for 2014–2015; and 112.02 ± 22.28 (56.01%) for 2015–2016. In all academic years, the interpretation for EC was more positive than negative. Although we observed a decrease in EC mean value, the only significant difference was observed in ‘Learning domain’ (*p* = 0.013) when academic year 2015–2016 was compared to 2010–2011 (*p* = 0.003) and 2014–2015 (*p* = 0.009) (Table 1). The average results obtained for each item with respect to the academic years are shown in Table 2 and Table 3. The following eight problematic items were common to all academic years (items 3, 4, 12, 13, 24, 25, 29, and 48): “There is a good support system for students who get stressed”, “I am too tired to enjoy this course”, “This school is well timetabled”, “The teaching is student-centred”, “The teaching time is put to good use”, “The teaching over-emphasises factual learning”, “The teachers are good at providing feedback to students”, and “The teaching is too teacher-centred”. On the other hand, only two positive items (15 and 46) were common to all academic years: “I have good friends at this school” and “My accommodation is pleasant”. In addition, comparing the results among the different academic years, statistically significant differences were observed in 17 items. Three items (18%) belonged to Learning domain (items 1, 7, and 25); another three items (8%) to Teaching domain (items 9, 37, and 40); five items (29%) to Academic domain (items 5, 10, 21, 31 and 45); one item (6%) to Atmosphere domain (item 12) and five items (29%) to Social domain (items 3, 4, 14, 19 and 46). In addition, 47% of these items presented statistically significant differences between courses, with a value ≤ 2 (problematic items) (Supplementary Table S2).

### 3.4. Analysis of ‘Educational Climate’ by Teaching Courses

In the 4th course of the 2014–2015 academic year, lower values were observed in all domains (except in the Teaching domain) compared to previous years, although differences were not significant. In addition, this 4th course corresponded to 5th course in the 2015–2016 academic year, where all domains showed significantly lower values in Learning domain as compared to academic years 2010–2011 (*p* = 0.001), 2013–2014 (*p* = 0.001), and 2014–2015 (*p* = 0.005. The overall EC also showed a lower value, although it was not significant (*p* = 0.057) (Table 4).

## 4. Discussion

To the best of our knowledge, the present study represents the first longitudinal analysis of EC, in a period of ‘curricular transition’ in Dentistry. EC is considered the expression and manifestation of the curriculum. It represents a critical element of the analysis of the quality of the teaching-learning process [2,24]. Our analysis of EC in different academic years and courses facilitates the detection of strengths and weaknesses from the student perspective and may contribute to strategies for educational improvement.

Although the DREEM survey evaluates the perception of teaching in five aspects of learning, it is not designed to analyse specific clinical or laboratory lessons, nor type of dental treatments performed by the students during their training. According to Miles et al. [25] the assessment of student percentages in the DREEM questionnaire provides a different analytical approach to comparing mean scores for overall scale, domains, and items. Keeping this in mind, we have expressed the results as both mean values and percentages. Most Health Science studies have reported EC values between 101–140 (51–70%) [4,9,26,27,28,29]. In the dental field, Zamzuri et al. [30] were the first to analyse EC for Dental Assistant and Dental Prosthesis Students from a Dental Training Institute in Malaysia, reporting 62.5% (125/200) and 59% (118/200), respectively. Subsequently, in a study involving 126 students from the Dentistry School of Manipal (India), Thomas et al. [16] found an EC mean of 57% (115/200). In our study, the result obtained for EC was 58% (115.70/200), which is interpreted by other authors as a more positive than negative perception [9,13,16,30,31]. However, higher EC values have been reported in studies conducted in New Zealand, Australia, and Germany [15,32,33]. This positive perception is in accordance with findings reported by members of our team in a multicenter study [17] performed at nine Spanish Public Schools of Dentistry. Our team performed a psychometric validation of the DREEM Spanish-language version involving 1391 students at the same Dentistry Schools. Results from this validation revealed that the Spanish version of the DREEM is a reliable and valid instrument for analysing the EC for dental students. These findings indicate that the DREEM is culturally independent [23].

In the present study, all domain values showed a percentage >52%, which were interpreted as “positive and acceptable”. The best score was for Social domain. However, Edgren et al. [34] stated that obtaining optimal results in the general perception of EC and its domains or subscales could mask the existence of specific problems. For this reason, it is very important to analyse the individual values of each questionnaire item [34,35]. We found four items scoring ≥3 in almost every academic year and interpreted as positive: item 15 (I have good friends in this school), item 46 (My accommodation is pleasant), item 10 (I am confident about my passing this year) and item 19 (My social life is good). Our findings are in line with Thomas et al. [16] for item 10 and with Kang et al. [14] for item 15. In our study, most of the positive items were associated with aspects of students’ social life. Like other authors [13,15,36,37], we found no excellent items, indicating that improvement measures must continue to be applied to our curriculum and educational environment. A total of forty-seven items (23.5%) were associated with problematic educational areas. The worst score was detected for item 3 (“There is a good support system for students who get stressed”), suggesting that a solution is needed, since stress may lead to worse academic outcomes [16]. In fact, in the study by Tomás et al. [38], item 3 was found to be a problematic aspect for both teachers and students in Spanish Schools of Dentistry. According to a number of studies, the lack of leisure time and anxiety associated with exams could be factors involved in stress [39,40,41,42]. It is widely observed that dental studies present high levels of stress associated with manifestations such as insomnia, eating disorders, inability to concentrate, hostility, and depression [43,44,45]. To improve this educational problem, Avalos et al. [46] proposed the implementation of a more individualized tutoring system and a student ‘mentoring’ program. Whittle et al. [47] advised the wider promotion and dissemination of existing university student support systems. At present, and in line with the educational reforms associated with the Bologna Process, these measures are being implemented in Spanish Schools of Dentistry. Apart from stress, we found another three negative items: “This school is well timetabled” (item 12), “The teaching over emphasizes factual learning” (item 25) and “The teaching is too teacher-centred” (item 48), which also received negative scores in the study by Ostapczuk et al. [32].

We found students’ perceptions of EC to be higher (59.01%) in the traditional curriculum (2010–2011) compared to the Bologna curriculum (2015–2016) (56.01%), although differences were not significant. This means that the development of the new curriculum did not have a significant negative impact on CE in its early years. Only Learning domain showed significant differences between 2010–2011 and 2015–2016 academic years. A higher percentage (30%) of items were identified as ‘problematic educational areas’ in the 2015–2016 academic year. While Tomás et al. [17] found a lower number of problematic items (14%), other authors reported problematic scores in 28% of items in the later academic years [16]. Of the fifteen total problematic items, eight (items 3, 4, 12, 13, 24, 25, 29, and 48) were present in all academic years and have also been reported by numerous other authors [13,15,36,37]. Unlike various studies [12,13,14,15], we did not find item 27 to be problematic, probably due to better memorization methods and more effective task management by students. Considering the problematic items present in all academic years, 50% were involved in the Learning domain (items 13, 24, 25, and 48). This tendency was also reported by Ahmad et al. [36].

With respect to courses and academic years, all domains presented higher values in the academic year 2010–2011. Interestingly, the Learning domain presented a value of 46.25% in the 4th course of the 2014–2015 academic year and 45.35% in the 5th course of the 2015–2016 academic year, reflecting a negative perception of the learning process in courses adapted to the Bologna education reform. Moreover, this domain was the only one that showed statistically significant differences between the 5th course in different academic years. Student perception in the final course is in line with the findings reported by other authors [33,37]. It may be related to the greater responsibility and need for self-learning associated with intensive clinical work.

One of the notable strengths of this study was its prospective longitudinal design. Nevertheless, the limitation that it was conducted at a single institution with a limited sample size should be kept in mind. This study was designed to reveal the problematic educational areas related to the idiosyncrasy of our own institution in order to improve several curricular aspects.

## 5. Conclusions

Overall, EC and its domains were perceived more positively than negatively by dental students during a period of ‘curricular transition’. The Social domain was the most positively evaluated, while the Learning domain was the worst. Our analysis revealed problematic educational areas during the transition from traditional to Bologna curricula, especially related to the Learning domain. The identification of problematic educational areas through the DREEM scale has potential for assessing the educational needs of higher education students to develop strategies for enhancing the teaching-learning process.

## Figures and Tables

**Table 1:** Mean values (%) of the ‘Educational Climate’ and the domains of the Dundee Ready Education Environment Measure (DREEM) questionnaire based on academic years.

DREEM	Global Mean (%)	2010–2011 Mean (%)	2013–2014 Mean (%)	2014–2015 Mean (%)	2015–2016 Mean (%)	*p*-Value *
Educational Climate (EC)	115.70 (57.85%)	118.02 (59.01%)	116.46 (58.23%)	115.60 (57.80%)	112.02 (56.01%)	0.178
Learning (D1)	25.12 (52.12%)	25.94 (54.04%)	25.19 (52.47%)	26.00 (54.1%)	23.31 (48.56%)	0.013
Teachers (D2)	25.15 (57.15%)	26.15 (59.43%)	24.58 (57.07%)	25.09 (55.86%)	24.60 (56%)	0.154
Academic (D3)	19.65 (61.40%)	19.96 (62.37%)	19.82 (61.93%)	19.32 (60.37%)	19.29 (60.28%)	0.663
Atmosphere (D4)	28.44 (59.25%)	28.30 (59.00%)	28.89 (60.18%)	28.57 (59.52%)	28.01 (58.35%)	0.422
Social (D5)	17.21 (61.49%)	17.66 (63.07%)	17.49 (62.46%)	16.60 (59.28%)	16.78 (59.92%)	0.112

* The comparison of the mean values of the items between all academic years was performed by applying the Kruskal-Wallis test.

**Table 2:** Mean values of the 50 items of the Dundee Ready Education Environment Measure (DREEM) questionnaire based on academic years.

Items	2010–2011 Mean	2013–2014 Mean	2014–2015 Mean	2015–2016 Mean
1. I am encouraged to participate in class	2.09	2.35	2.58	2.11
2. The teachers are knowledgeable	2.75	2.61	2.7	2.82
3. There is a good support system for students who get stressed	**0.94**	**1.14**	**1.42**	**0.9**
*4. I am too tired to enjoy the course*	**1.68**	**1.83**	**1.48**	**1.35**
5. Learning strategies which worked for me before continue to work for me now	2.58	2.54	2.19	2.56
6. The teachers are patient with patients	2.45	2.37	2.27	2.36
7. The teaching is often stimulating	2.22	**1.87**	2.16	**1.83**
*8. The teachers ridicule the students*	2.54	2.28	2.23	2.16
*9. The teachers are authoritarian*	**1.67**	**1.56**	2.01	**1.55**
10. I am confident about my passing this year	2.88	3.28	3.1	3.05
11. The atmosphere is relaxed during the ward teaching	2.33	2.23	2.3	2.14
12. This school is well timetabled	**1.13**	**1.67**	**1.55**	**1.19**
13. The teaching is student-centred	**1.77**	**1.74**	**1.71**	**1.57**
14. I am rarely bored on this course	2.27	**1.88**	2.18	**1.86**
15. I have good friends in this school	3.43	3.45	3.12	3.35
16. The teaching helps to develop my competence	2.72	2.75	2.62	2.62
*17. Cheating is a problem in this school*	2.78	2.48	2.74	2.52
18. The teachers have good communications skills with patients	2.74	2.81	2.53	2.56
19. My social life is good	3.17	3.02	2.75	3.14
20. The teaching is well focused	2.03	**1.96**	2.08	**1.72**
21. I feel I am being well prepared for my profession	**1.94**	**1.68**	2.01	**1.44**
22. The teaching helps to develop my confidence	2.19	2.06	2.18	**1.89**
23. The atmosphere is relaxed during lectures	2.42	2.45	2.44	2.3
24. The teaching time is put to good use	**1.78**	**1.9**	**1.95**	**1.64**
*25. The teaching overemphasizes factual learning*	**1.68**	**1.68**	**1.78**	**0.88**
26. Last year’s work has been good preparation for this year’s work	**1.9**	2.11	2.08	2.4
27. I am able to memorize all I need	2.39	2.3	2.41	2.4
28. I seldom feel lonely	2.77	2.71	2.55	2.79
29. The teachers are good at providing feedback to students	**1.93**	**1.93**	**2.00**	**1.7**
30. There are opportunities for me to develop interpersonal skills	2.36	2.55	2.34	2.6
31. I have learned a lot about empathy in my profession	2.71	2.92	2.53	2.7
32. The teachers provide constructive criticism here	2.42	2.21	2.21	2.05
33. I feel comfortable in class socially	2.94	3.03	2.82	3.16
34. The atmosphere is relaxed during seminars/tutorials	2.71	2.65	2.66	2.72
*35. I find the experience disappointing*	2.49	2.57	2.38	2.23
36. I am able to concentrate well	2.57	2.65	2.49	2.71
37. The teachers give clear examples	2.55	2.21	2.21	2.32
38. I am clear about the learning objectives of the course	2.72	2.57	2.42	2.7
*39. The teachers get angry in class*	2.17	2.06	2.18	2.07
40. The teachers are well prepared for their classes	2.49	2.21	2.22	2.57
41. My problem-solving skills are being well developed here	2.63	2.47	2.59	2.38
42. The enjoyment outweighs the stress of the course	2.18	2.05	2.22	1.98
43. The atmosphere motivates me as a learner	2.27	2.27	2.44	2.11
44. The teaching encourages me to be an active learner	2.45	2.3	2.45	2.21
45. Much of what I have to learn seems relevant to a career in health care	2.93	2.61	2.41	2.76
46. My accommodation is pleasant	3.43	3.38	3.1	3.39
47. Long-term learning is emphasized over short term learning	2.68	2.42	2.48	2.6
*48. The teaching is too teacher-centred*	**1.62**	**1.46**	**1.59**	**1.54**
49. I feel able to ask the questions I want	2.13	2.34	2.19	2.35
50. The students irritate the teachers	2.45	2.21	2.53	2.43

The items (4, 8, 9, 17, 25, 35, 39, 48, and 50) in cursive are negative statements and their scores were reversed. Item with an average of ≤2 are in bold. Items with an average of >3 are underlined.

**Table 3:** Number, percentage (%) and interpretation of items on the Dundee Ready Education Environment Measure (DREEM) questionnaire by academic years.

Interpretation of Individual Items	2010–2011 (%)	2013–2014 (%)	2014–2015 (%)	2015–2016 (%)
≤2.00 = Educational problematic areas, which should be examined more exhaustively later	11 (22%)	13 (26%)	8 (16%)	15 (30%)
2.01–3.00 = Educational aspects that could be improved	36 (72%)	32 (64%)	39 (78%)	30 (60%)
3.01–3.49 = Positive educational aspects	3 (6%)	5 (10%)	3 (6%)	5 (10%)
≥3.50 = Educational aspects of excellence	0 (0%)	0 (0%)	0 (0%)	0 (0%)

**Table 4:** Mean values of the ‘Educational Climate’ and the domains of the Dundee Ready Education Environment Measure (DREEM) questionnaire of 3rd, 4th and 5th courses on the different academic years.

	2010–2011 Mean ± SD (%)	2013–2014 Mean ± SD (%)	2014–2015 Mean ± SD (%)	2015–2016 Mean ± SD (%)	*p*-Value *
*3rd course*					
**D1**	26.38 ± 4.73 (62.80%)	24.12 ± 7.30 (50.25%)	28.24 ± 4.57 (58.83%)	24.85 ± 7.59 (51.77%)	0.097
**D2**	27.87 ± 4.84 (63.34%)	24.84 ± 7.18 (56.45%)	25.88 ± 5.33 (58.81%)	25.53 ± 5.46 (58.02%)	0.222
**D3**	19.38 ± 3.04 (60.58%)	19.51 ± 4.39 (60.96%)	19.72 ± 4.26 (61.62%)	18.14 ± 4.68 (56.68%)	0.588
**D4**	29.54 ± 5.38 (61.54%)	27.66 ± 6.87 (57.62%)	28.16 ± 5.24 (58.66%)	28.46 ± 6.17 (59.29%)	0.690
**D5**	17.80 ± 3.19 (63.57%)	16.87 ± 3.49 (60.25%)	16.08 ± 3.49 (57.42%)	16.53 ± 4.25 (59.03%)	0.300
**EC**	121.00 ± 15.95 (60.50%)	115.06 ± 21.41 (57.53%)	118.08 ± 19.93 (59.04%)	113.53 ± 24.39 (56.76%)	0.481
*4th course*					
**D1**	25.19 ± 4.66 (52.47%)	24.33 ± 5.83 (50.68%)	22.20 ± 7.09 (46.25%)	24.28 ± 4.90 (50.58%)	0.325
**D2**	26.10 ± 5.21 (59.31%)	23.46 ± 4.93 (53.31%)	25.45 ± 5.05 (55.56%)	26.14 ± 5.75 (59.40%)	0.181
**D3**	19.50 ± 3.2 (60.93%)	18.86 ± 4.58 (58.93%)	17.10 ± 4.06 (53.43%)	19.28 ± 4.30 (60.25%)	0.116
**D4**	27.32 ± 5.5 (56.91%)	28.22 ± 6.09 (58.79%)	26.35 ± 6.81 (54.89%)	28.47 ± 5.14 (59.31%)	0682
**D5**	17.17 ± 3.45 (61.31%)	16.36 ± 4.23 (58.42%)	16.00 ± 3.30 (57.14%)	17.19 ± 2.89 (61.39%)	0.631
**EC**	115.30 ± 17.15 (57.65%)	112.00 ± 20.72 (60.00%)	107.10 ± 20.58 (53.55%)	115.38 ± 18.40 (57.69%)	0.558
*5th course*					
**D1**	26.41 ± 4.90 (55.02%)	26.59 ± 5.20 (55.39%)	26.71 ± 6.07 (55.64%)	21.77 ± 6.94 (45.35%)	0.001 **
**D2**	24.95 ± 5.87 (5670%)	25.13 ± 6.46 (57.11%)	24.14 ± 5.5 (54.86%)	23.40 ± 5.8 (53.18%)	0.464
**D3**	20.88 ± 3.60 (65.25%)	20.68 ± 3.63 (64.62%)	20.57 ± 4.8 (64.28%)	19.95 ± 4.4 (62.34%)	0.566
**D4**	28.44 ± 5.53 (59.25%)	30.26 ± 5.24 (63.04%)	30.53 ± 6.57 (63.60%)	27.43 ± 5.77 (57.14%)	0.064
**D5**	18.09 ± 2.74 (64.60%)	18.65 ± 3.09 (66.60%)	17.50 ± 4.54 (62.50%)	16.65 ± 3.75 (59.46%)	0.123
**EC**	118.79 ± 18.53 (59.39%)	120.68 ± 17.3 (60.34%)	119.46 ± 23.6 (59.73%)	109.22 ± 22.9 (53.61%)	0.057

* The comparison of the mean values of the items between all academic years was performed by applying the Kruskal–Wallis test. ** *p*-Value < 0.05.

## Supplementary Materials

The following are available online at https://www.mdpi.com/2304-6767/8/4/133/s1, Table S1: Dundee Ready Education Environment (DREEM) questionnaire (50 items). Table S2: Mean values of the items with statistically significant differences with respect to the academic years.
