# Supplementary material for: Students’ Perceptions of Educational Climate in a Spanish School of Dentistry Using the Dundee Ready Education Environment Measure: A Longitudinal Study
Source: Dent J (Basel). 2020 Dec 7;8(4):133. doi: 10.3390/dj8040133 (PMC7762267; doi:10.3390/dj8040133)
Supplement: Supplementary file 1 [file dentistry-08-00133-s001.pdf]

Students' Perceptions of Educational Climate in a Spanish School of Dentistry using the Dundee Ready Education Environment Measure: a longitudinal study

Hernández-Crespo Alba María, Fernández-Riveiro Paula, Rapado-González Óscar, Aneiros Angela, Tomás Inmaculada, Suárez-Cunqueiro María Mercedes

Supplementary Material

Table S1. Dundee Ready Education Environment (DREEM) questionnaire (50 items).

ITEMS	RESPONSES				
1. I am encouraged to participate in class	SA	A	U	D	SD
2. The teachers are knowledgeable	SA	A	U	D	SD
3. There is a good support system for students who get stressed	SA	A	U	D	SD
4. I am too tired to enjoy the course	SA	A	U	D	SD
5. Learning strategies which worked for me before continue to work for me now	SA	A	U	D	SD
6. The teachers are patient with patients	SA	A	U	D	SD
7. The teaching is often stimulating	SA	A	U	D	SD
8. The teachers ridicule the students	SA	A	U	D	SD
9. The teachers are authoritarian	SA	A	U	D	SD
10. I am confident about my passing this year	SA	A	U	D	SD
11. The atmosphere is relaxed during the ward teaching	SA	A	U	D	SD
12. This school is well timetabled	SA	A	U	D	SD
13. The teaching is student-centred	SA	A	U	D	SD
14. I am rarely bored on this course	SA	A	U	D	SD
15. I have good friends in this school	SA	A	U	D	SD
16. The teaching helps to develop my competence	SA	A	U	D	SD
17. Cheating is a problem in this school	SA	A	U	D	SD
18. The teachers have good communications skills with patients	SA	A	U	D	SD
19. My social life is good	SA	A	U	D	SD
20. The teaching is well focused	SA	A	U	D	SD
21. I feel I am being well prepared for my profession	SA	A	U	D	SD
22. The teaching helps to develop my confidence	SA	A	U	D	SD
23. The atmosphere is relaxed during lectures	SA	A	U	D	SD
24. The teaching time is put to good use	SA	A	U	D	SD
25. The teaching overemphasises factual learning	SA	A	U	D	SD
26. Last year's work has been a good preparation for this year's work	SA	A	U	D	SD
27. I am able to memorise all I need	SA	A	U	D	SD
28. I seldom feel lonely	SA	A	U	D	SD
29. The teachers are good at providing feedback to students	SA	A	U	D	SD
30. There are opportunities for me to develop interpersonal skills	SA	A	U	D	SD
31. I have learned a lot about empathy in my profession	SA	A	U	D	SD
32. The teachers provide constructive criticism here	SA	A	U	D	SD
33. I feel comfortable in class socially	SA	A	U	D	SD
34. The atmosphere is relaxed during seminars/tutorials	SA	A	U	D	SD
35. I find the experience disappointing	SA	A	U	D	SD
36. I am able to concentrate well	SA	A	U	D	SD

ITEMS	RESPONSES				
37. The teachers give clear examples	SA	A	U	D	SD
38. I am clear about the learning objectives of the course	SA	A	U	D	SD
39. The teachers get angry in class	SA	A	U	D	SD
40. The teachers are well prepared for their classes	SA	A	U	D	SD
41. My problem-solving skills are being well developed here	SA	A	U	D	SD
42. The enjoyment outweighs the stress of the course	SA	A	U	D	SD
43. The atmosphere motivates me as a learner	SA	A	U	D	SD
44. The teaching encourages me to be an active learner	SA	A	U	D	SD
45. Much of what I have to learn seems relevant to a career in health care	SA	A	U	D	SD
46. My accommodation is pleasant	SA	A	U	D	SD
47. Long-term learning is emphasised over short term learning	SA	A	U	D	SD
48. The teaching is too teacher centred	SA	A	U	D	SD
49. I feel able to ask the questions I want	SA	A	U	D	SD
50. The students irritate the teachers	SA	A	U	D	SD

SA, strongly agree; A, agree; U, uncertain; D, disagree; SD, strongly disagree.

Table S2. Mean values of the items with statistically significant differences respect to the academic years.

Items	2010-11 Mean ± SD	2013-14 Mean ± SD	2014-15 Mean ± SD	2015-16 Mean ± SD	*p-value**
1. I am encouraged to participate in class	2.09 ± 2.00	2.35 ± 0.94	2.58 ± 1.04	2.11 ± 1.04	0.002
3. There is a good support system for students who get stressed	0.94 ± 1.00	1.14 ± 0.94	1.42 ± 1.18	0.90 ± 0.97	0.008
4. I am too tired to enjoy the course	1.68 ± 1.13	1.83 ± 1.24	1.48 ± 1.10	1.35 ± 1.12	0.023
5. Learning strategies which worked for me before continue to work for me now	2.58 ± 0.98	2.54 ± 1.06	2.19 ± 1.06	2.56 ± 1.27	0.026
7. The teaching is often stimulating	2.22 ± 0.86	1.87 ± 1.02	2.16 ± 0.98	1.83 ± 1.05	0.020
9. The teachers are authoritarian	1.67 ± 0.97	1.56 ± 0.96	2.01 ± 1.14	1.55 ± 0.99	0.022
10. I am confident about my passing this year	2.88 ± 0.79	3.28 ± 0.63	3.10 ± 0.80	3.05 ± 0.83	0.002
12. This school is well timetabled	1.13 ± 0.96	1.67 ± 1.05	1.55 ± 1.20	1.19 ± 1.08	0.000
14. I am rarely bored on this course	2.27 ± 1.00	1.88 ± 1.21	2.18 ± 1.12	1.86 ± 1.15	0.017
19. My social life is good	3.17 ± 0.84	3.02 ± 0.93	2.75 ± 1.03	3.14 ± 0.78	0.018
21. I feel I am being well prepared for my profession	1.94 ± 0.99	1.68 ± 1.10	2.01 ± 1.06	1.44 ± 1.04	0.018

25. The teaching overemphasizes factual learning	1.68 ± 0.93	1.68 ± 1.04	1.78 ± 1.09	0.88 ± 0.78	0.000
31. I have learned a lot about empathy in my profession	2.71 ± 1.02	2.92 ± 0.80	2.53 ± 1.00	2.70 ± 0.90	0.039
37. The teachers give clear examples	2.55 ± 0.74	2.21 ± 0.86	2.21 ± 0.84	2.32 ± 0.85	0.012
40. The teachers are well prepared for their classes	2.49 ± 0.93	2.21 ± 0.95	2.22 ± 0.94	2.57 ± 0.66	0.013
45. Much of what I have to learn seems relevant to a career in health care	2.93 ± 0.84	2.61 ± 0.95	2.41 ± 1.02	2.76 ± 0.92	0.001
46. My accommodation is pleasant	3.43 ± 0.79	3.38 ± 0.83	3.10 ± 0.81	3.39 ± 0.62	0.009

*The comparison of the mean values of the items between all academic years was performed by applying the Kruskal-Wallis test.
